# OD18 Surrogate Measures And The Health Technology Assessment Of Cancer Drugs In Ireland: A Retrospective Analysis, 2017 to 2022

**DOI:** 10.1017/S0266462324001521

**Published:** 2025-01-07

**Authors:** Alexandre Assi, Tereza Karbanova, Lama Algawas, Yue Xiang Darren Lim, Lea Trela-larsen, Cara Usher, Michael Barry, Caroline Walsh

## Abstract

**Introduction:**

Surrogate endpoints are increasingly being used in the pivotal trials of cancer drugs to underpin (conditional) regulatory approval. We examined the relationship between the use of surrogate measures in pivotal trials underpinning cancer drug approvals by the European Medicines Agency (EMA) between 2017 and 2022 and health technology assessment (HTA) recommendations made by the National Centre for Pharmacoeconomics in Ireland (NCPE).

**Methods:**

A previously published methodology was used to identify cancer drug indications that received (conditional) marketing authorization between 2017 and 2022, inclusive. EMA-approved cancer drugs were categorized using the following benefit categories, based on pivotal trial endpoints: overall survival (OS), progression-free survival (PFS), disease response (DR), and single-arm trials (SATs). The NCPE website was searched to identify indications that had undergone, at least, a rapid review (RR) assessment. The NCPE recommendation for each assessment was recorded. Additional data including the incremental quality-adjusted life years (QALY) gain reported in cost-effectiveness analyses were extracted for indications that had undergone a full HTA.

**Results:**

One hundred and eight cancer drug indications were identified, comprising 68 cancer drugs. In 2017, OS, PFS, and SAT benefit underpinned equal proportions of approvals (28.6% each). In 2022, SAT underpinned the largest proportion of approvals (53.6%). As of June 2023, 77 indications (71.3%) had undergone at least a RR assessment; 31 indications had completed a full HTA appraisal. All of the indications underpinned by SAT evidence (n=7) received a conditional negative recommendation. Indications with SAT evidence had a mean incremental QALY gain of 1.88 (standard deviation [SD] 1.20), whereas indications with an OS benefit had a mean incremental QALY gain of 0.81 (SD 0.36).

**Conclusions:**

The proportion of cancer drug indications receiving regulatory approval on the basis of SAT evidence, where no direct comparative evidence is available, is increasing. This results in additional uncertainty in the comparative benefit of cancer drugs supported by SAT evidence. The study is limited by the sample size of HTA appraisals included. Further in-depth analysis of factors influencing NCPE recommendations is needed.

